# Adequação do Consumo de Ácidos Graxos entre Pacientes em Prevenção Cardiovascular Secundária

**DOI:** 10.36660/abc.20230487

**Published:** 2024-03-20

**Authors:** Aline Marcadenti, Rachel H. Vieira Machado, Renato Hideo Nakagawa Santos, Caio Cesar dos Santos Kasai, Cristiane Kovacs, Annie Bello, Cristina H. de Matos, Renata Torres Abib Bertacco, Gabriela C. Souza, Gabriela da S. Schirmann, Francisca Eugenia Zaina Nagano, Soraia Poloni, Raquel Milani El Kik, Naoel Hassan Feres, Isa G. Rodrigues, Antônio Carlos Sobral Sousa, Josilene M. F. Pinheiro, Sandra Mary Lima Vasconcelos, Daniele Maria de Oliveira Carlos, Viviane Sahade Souza, Adriana Barros Gomes, José Albuquerque de Figueiredo, Emilio Hideyuki Moriguchi, Maria Cristina Izar, Sônia Lopes Pinto, Josefina Bressan, Simone Raimondi de Souza, Magali C. Kumbier, Celme Barroncas Passos de Araújo, Camila R. Torreglosa, Bernardete Weber, Ângela Cristine Bersch-Ferreira

**Affiliations:** 1 Instituto de Pesquisa do Hcor São Paulo SP Brasil Instituto de Pesquisa do Hcor, São Paulo, SP – Brasil; 2 Pontifícia Universidade Católica do Paraná Escola de Medicina Câmpus Londrina Londrina PR Brasil Pontifícia Universidade Católica do Paraná – Escola de Medicina Câmpus Londrina, Londrina, PR – Brasil; 3 Instituto Dante Pazzanese de Cardiologia São Paulo SP Brasil Instituto Dante Pazzanese de Cardiologia, São Paulo, SP – Brasil; 4 Instituto Nacional de Cardiologia Rio de Janeiro RJ Brasil Instituto Nacional de Cardiologia, Rio de Janeiro, RJ – Brasil; 5 Universidade do Vale do Itajaí Itajaí SC Brasil Universidade do Vale do Itajaí, Itajaí, SC – Brasil; 6 Universidade Federal de Pelotas Pelotas RS Brasil Universidade Federal de Pelotas, Pelotas, RS – Brasil; 7 Universidade Federal do Rio Grande do Sul Porto Alegre RS Brasil Universidade Federal do Rio Grande do Sul, Porto Alegre, RS – Brasil; 8 Universidade da Região da Campanha Bagé RS Brasil Universidade da Região da Campanha, Bagé, RS – Brasil; 9 Universidade Federal do Paraná Hospital de Clínicas Curitiba PR Brasil Universidade Federal do Paraná – Hospital de Clínicas, Curitiba, PR – Brasil; 10 Hospital de Clínicas de Porto Alegre Porto Alegre RS Brasil Hospital de Clínicas de Porto Alegre, Porto Alegre, RS – Brasil; 11 Pontifícia Universidade Católica do Rio Grande do Sul Porto Alegre RS Brasil Pontifícia Universidade Católica do Rio Grande do Sul, Porto Alegre, RS – Brasil; 12 Universidade Federal de Mato Grosso Cuiabá MT Brasil Universidade Federal de Mato Grosso, Cuiabá, MT – Brasil; 13 Pronto Socorro Cardiológico Universitário de Pernambuco Recife PE Brasil Pronto Socorro Cardiológico Universitário de Pernambuco, Recife, PE – Brasil; 14 Universidade Federal de Sergipe Aracaju SE Brasil Universidade Federal de Sergipe, Aracaju, SE – Brasil; 15 Hospital Universitário Ana Bezerra Santa Cruz RN Brasil Hospital Universitário Ana Bezerra, Santa Cruz, RN – Brasil; 16 Universidade Federal de Alagoas Maceió AL Brasil Universidade Federal de Alagoas, Maceió, AL – Brasil; 17 Hospital de Messejana Fortaleza CE Brasil Hospital de Messejana, Fortaleza, CE – Brasil; 18 Universidade Federal da Bahia Salvador BA Brasil Universidade Federal da Bahia, Salvador, BA – Brasil; 19 Universidade Federal do Maranhão São Luís MA Brasil Universidade Federal do Maranhão, São Luís, MA – Brasil; 20 Universidade Federal de São Paulo São Paulo SP Brasil Universidade Federal de São Paulo (UNIFESP), São Paulo, SP – Brasil; 21 Universidade Federal do Tocantins Palmas TO Brasil Universidade Federal do Tocantins, Palmas, TO – Brasil; 22 Universidade Federal de Viçosa Viçosa MG Brasil Universidade Federal de Viçosa, Viçosa, MG – Brasil; 23 Instituto Estadual de Cardiologia Aloysio de Castro Rio de Janeiro RJ Brasil Instituto Estadual de Cardiologia Aloysio de Castro, Rio de Janeiro, RJ – Brasil; 24 Consultoria Terapia Nutricional Porto Alegre RS Brasil Consultoria Terapia Nutricional – COTENUT, Porto Alegre, RS – Brasil; 25 Hospital Universitário Francisca Mendes Manaus AM Brasil Hospital Universitário Francisca Mendes, Manaus, AM – Brasil; 26 Real e Benemérita Associação Portuguesa de Beneficência São Paulo SP Brasil Real e Benemérita Associação Portuguesa de Beneficência, São Paulo, SP – Brasil

**Keywords:** Ácidos Graxos, Dieta, Doenças Cardiovasculares, Prevenção Secundária

## Abstract

**Fundamento:**

A adesão à uma alimentação adequada em macronutrientes é fundamental para a prevenção secundária de doenças cardiovasculares.

**Objetivo:**

Avaliar a prevalência de adesão às recomendações de consumo de ácidos graxos para prevenção e tratamento de doenças cardiovasculares, e estimar se a presença de determinados fatores de risco cardiovascular estaria associada à adesão.

**Métodos:**

Estudo transversal com os dados de linha de base de 2358 participantes do estudo *"Brazilian Cardioprotective Nutritional Program Trial"* . Dados de consumo alimentar, e fatores de risco cardiovascular foram avaliados. Foi considerada, de acordo com a Sociedade Brasileira de Cardiologia, uma ingestão adequada de ácidos graxos poli-insaturados (AGPI) ≥10% do consumo total de energia diária, para ácidos graxos monoinsaturados (AGM), 20% e para ácidos graxos saturados (AGS), <7%. Na análise estatística foi considerando nível de significância de 5%.

**Resultados:**

Nenhum participante aderiu a todas as recomendações de forma simultânea e mais da metade (1482 [62,9%]) não aderiu a nenhuma recomendação. A adesão exclusivamente à recomendação de AGS foi a mais prevalente, sendo cumprida por 659 (28%) dos participantes, seguida da adesão exclusivamente à recomendação de AGP (178 [7,6%]) e de AGM (5 [0,2%]). Não houve associação entre o número de comorbidades e a adesão às recomendações nutricionais (p =0,269). Os participantes da região Nordeste do país apresentaram maior proporção de adesão às recomendações para consumo de AGS (38,42%), e menor para ingestão de AGPI (3,52%) (p <0,001) em comparação às demais.

**Conclusões:**

Na amostra avaliada, evidenciou-se baixa adesão às recomendações nutricionais para consumo de ácidos graxos.

**Figure:**
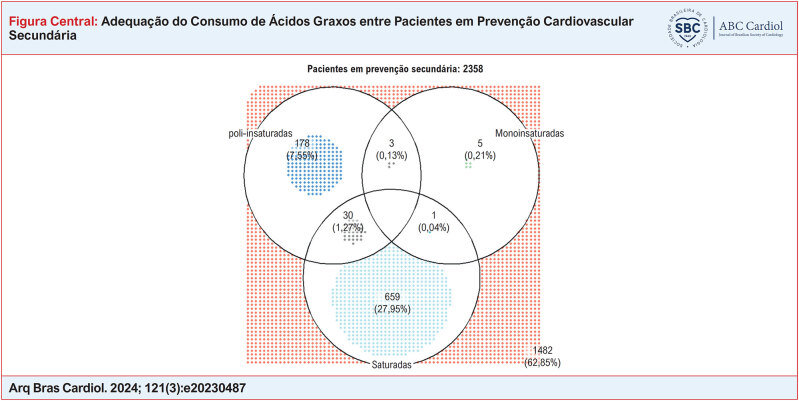


## Introdução

As doenças cardiovasculares (DCV) são consideradas a principal causa de morbimortalidade no Brasil e no mundo, sendo responsáveis por cerca de sete milhões de mortes por ano, principalmente em grupos vulneráveis, como idosos, pessoas de menor renda e baixa escolaridade.^1 , 2^

Os fatores de risco para as DCV são alvo de prevenção para a ocorrência dessas doenças. Porém, atenção maior ao tratamento deve ser dada aos indivíduos em prevenção cardiovascular secundária, uma vez que é estabelecido maior risco para recorrência de eventos nesta população.^3 , 4^ Esses fatores de risco podem ser modificáveis ou não. Os fatores modificáveis incluem hiperlipidemia, tabagismo, consumo de álcool, hiperglicemia, obesidade, sedentarismo, hipertensão arterial sistêmica (HAS) e má qualidade da dieta.^5^

Diretrizes clínicas referentes a abordagem nutricional são desenvolvidas por sociedades médicas para orientar o consumo de nutrientes e padrões alimentares relacionados a proteção e ao risco para o desenvolvimento de DCV.^6^ Uma das recomendações nutricionais em destaque nas diretrizes é referente à importância da proporção da ingestão de diferentes ácidos graxos: saturados (AGS), monoinsaturados (AGM), poli-insaturados (AGPI) e trans-insaturados (AGT).^7 - 9^ As diretrizes recomendam que os profissionais de saúde orientem seus pacientes a terem um consumo alimentar seguindo padrões dietéticos saudáveis incluindo uma proporção adequada de ácidos graxos; entretanto, não descrevem como essa orientação deve ser realizada. Além disso, é necessário adaptar essas orientações para o contexto cultural e financeiro nos quais o indivíduo se encontra.^10 , 11^

A relação entre o consumo de diferentes ácidos graxos, principalmente AGS, com a ocorrência de eventos cardiovasculares na população em geral ainda não está estabelecida.^12 , 13^ No que diz respeito à prevenção cardiovascular secundária, pouco se sabe sobre o consumo desses nutrientes^14 , 15^ e informações nacionais em grande escala sobre a adesão desses pacientes da comunidade (não hospitalizados) às recomendações de consumo de ácidos graxos não são conhecidas. Assim, o objetivo do presente estudo foi avaliar, na linha de base de um ensaio clínico randomizado multicêntrico nacional, a prevalência de adesão às recomendações de consumo de ácidos graxos, bem como estimar se a presença de determinados fatores de risco cardiovascular (dislipidemia, HAS ou diabetes mellitus tipo 2 [DM2]) estaria associada à adesão.

## Métodos

Trata-se de uma análise exploratória dos dados de linha de base dos participantes do estudo " *Brasilian Cardioprotective Nutritional Program Trial - (BALANCE Program Trial. ClinTrials:* NCT01620398). Este foi um estudo multicêntrico, com a participação de 2534 indivíduos em prevenção secundária para doenças cardiovasculares provenientes de 35 centros distribuídos entre as 5 regiões brasileiras.^16^ O objetivo do ensaio clínico randomizado foi avaliar a efetividade de uma intervenção nutricional educativa, pautada nas recomendações nutricionais preconizadas pelas diretrizes brasileiras para prevenção das DCV, sobre a prevenção de novos eventos cardiovasculares.^16^

Todos os critérios de elegibilidade estão relatados no protocolo do estudo.^17^ Foram incluídos participantes com 45 anos ou mais que apresentaram um ou mais dos indicadores de DCV estabelecida nos últimos 10 anos: doença coronariana, acidente vascular cerebral, e doença vascular periférica. Todos os voluntários leram e assinaram o termo de consentimento antes de sua seleção final como sujeitos de pesquisa. O protocolo do estudo foi aprovado pelos Comitês de Ética locais.

### Coleta de dados

Entrevistadores treinados aplicaram um questionário estruturado contendo questões sobre características sociodemográficas^18^ e clínicas. Os participantes foram classificados como fumantes atuais, não fumantes ou ex-fumantes, e o nível de atividade física foi classificado de acordo com o questionário *International Physical Activity Questionnaire* (IPAQ) versão curta.^19^ O índice de massa corporal (IMC) foi calculado de acordo com a fórmula massa corporal (kg)/altura (m^2^). A pressão arterial foi obtida por um profissional treinado, seguindo as recomendações da *American Heart Association* ,^20^ e os dados referentes ao uso de medicações foram obtidos a partir de prescrições médicas. Os diagnósticos de DM2, HAS e dislipidemia foram obtidos dos prontuários. Todos os dados foram registrados em um formulário eletrônico de relato de caso ( *case report form* ).

### Consumo alimentar

Os dados de consumo alimentar foram obtidos por meio de dois recordatórios alimentares de 24 horas, sendo a média entre os dois recordatórios utilizada para a análise proposta. Participantes com relato de consumo alimentar >4000 kcal/dia ou < 500 kcal/dia (separar kcal/dia de 500) foram excluídos da análise por representarem possível relato inconsistente. O método de passagem múltipla ( *Multiple Pass Method* ) foi usado para padronizar a coleta de dados de consumo alimentar e permitir a captura do máximo de informações sobre os alimentos consumidos.^21^ Os dados de consumo alimentar foram registrados no programa Vivanda (São Paulo, SP, Brasil), software brasileiro que prioriza tabelas brasileiras e americanas de composição de alimentos.^22 , 23^ Um álbum de fotos contendo imagens de porções padronizadas de alimentos, especificamente preparado pelo BALANCE Program Trial, foi utilizado para auxiliar na avaliação da ingestão alimentar. Todos os pesquisadores envolvidos na coleta dos dados foram treinados tanto para a obtenção das informações quanto para a utilização do software.

Para verificar se o participante atingiu a recomendação de AGPI, foi considerada uma ingestão maior ou igual a 10% do consumo total de energia diária (CTE).^7 , 8^ O participante foi considerado como ter atingido a recomendação de ingestão de AGM caso tivesse reportado consumo maior ou igual a 20% do CTE.^8^ Por fim, para verificar se o sujeito atingiu a recomendação de AGS, foi considerada uma ingestão menor ou igual a 7% do CTE.^8 , 9^

### Análise estatística

Os dados foram apresentados em frequências absolutas e relativas para as variáveis categóricas e estatísticas de posição (média ou mediana) e de dispersão (desvio padrão e intervalos interquartis) para as variáveis contínuas, conforme normalidade dos dados avaliada pelo teste de Shapiro-Wilk. As comparações entre os participantes que atingiram ao menos uma das recomendações foram realizadas através do teste qui-quadrado de Pearson ou teste exato de Fisher quando apropriado, para as variáveis categóricas e teste t-student não pareado para as variáveis contínuas. Para a ingestão de macronutrientes os pacientes que atingiram uma determinada recomendação (ingestão de AGS, AGM e AGPI) foram comparados por meio do teste não paramétrico de Mann-Whitney. O diagrama de Venn foi utilizado para representar graficamente a ocorrência da adesão simultânea das recomendações de ingestão dos ácidos graxos. Todas as análises consideraram um alfa bicaudal de 5% e foram realizadas com o auxílio do software R (R foundation for Statistical Computing, Vienna, Austria).

## Resultados

**Figura 1:**
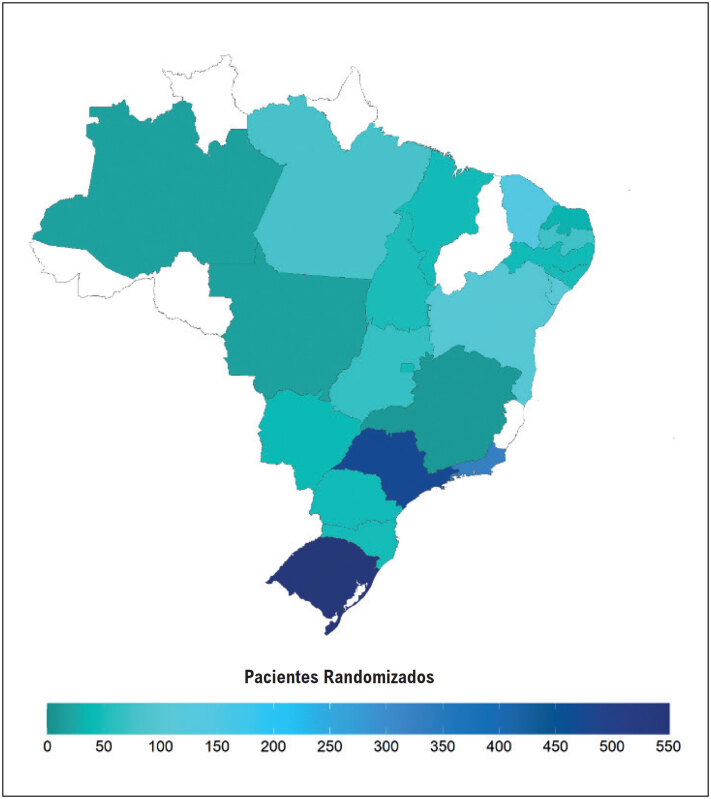
Distribuição dos participantes incluídos no estudo BALANCE por estado nacional.

No estudo BALANCE, 2534 indivíduos em prevenção secundária para doença cardiovascular foram incluídos; porém, para a presente análise, foram considerados apenas aqueles com registro alimentar completo. Participantes com relato de consumo alimentar >4000kcal ou < 500kcal/dia foram excluídos por representarem possível relato inconsistente do participante. Assim, a amostra total do estudo foi composta por 2358 indivíduos. Destes, 144 (6,1%) eram da região Norte do país, 169 (7,2%) da região Centro-Oeste, 596 (25,3%) da região Nordeste, 639 (27,1%) da região Sul e 810 (34,5%) da região Sudeste ( Figura 1 ).

Com relação às porcentagens medianas de ingestão dos ácidos graxos, identificou-se 8,7% (intervalo interquartil 6,6 - 10,7) de AGS, 7,8% (6,1 - 9,7) de AGM e 6,3% (4,9 – 8,0) de AGPI. Nenhum indivíduo aderiu totalmente as recomendações de consumo de ácidos graxos e mais da metade (1482 [62,9%]) não aderiu a nenhuma recomendação (Figura Central). A adesão exclusivamente à recomendação de AGS foi a mais prevalente, sendo cumprida por 659 (28%) participantes, seguida da adesão exclusivamente à recomendação de AGPI (178 [7,6%]) e de AGM (5 [0,2%]). Ainda, 30 indivíduos (1,3%) adeririam às recomendações de AGPI e AGS de forma concomitante, três indivíduos à recomendação de AGPI e AGM (0,1%) e apenas um indivíduo (0,04%) à recomendação de AGM e AGS simultaneamente.

Na Tabela 1 estão descritas as características dos participantes que aderiam a pelo menos uma recomendação (AGPI, AGM ou AGS) e daqueles que não aderiam a nenhuma das recomendações. Destaca-se que indivíduos de classes sociais inferiores pelo critério ABEP e com menor escolaridade apresentaram maior adesão às recomendações.

**Tabela 1:** Característica dos participantes que aderiam a alguma recomendação nutricional para consumo de ácidos graxos e daqueles que não aderiam a nenhuma recomendação

Características		Não adere a nenhuma recomendação (n=1482)	Adere a pelo menos 1 recomendação (n=876)	Total (n=2358)	p
**Dados demográficos**					
Idade (anos) - média ± dp		63,3 ± 9,1	62,9 ± 8,7 (n=876)	63,2 ± 9	0,259
Sexo Feminino		606/1482 (40,9%)	374/876 (42,7%)	980/2358 (41,6%)	0,411
**Classe social**					
	D/E	175/1375 (12,7%)	128/787 (16,3%)	303/2162 (14%)	**0,014**
	C	778/1375 (56,6%)	455/787 (57,8%)	1233/2162 (57%)	
	A/B	422/1375 (30,7%)	204/787 (25,9%)	626/2162 (29%)	
**Escolaridade**					
	Ensino fundamental	803/1375 (58,4%)	515/791 (65,1%)	1318/2166 (60,8%)	**0,004**
	Ensino médio	451/1375 (32,8%)	228/791 (28,8%)	679/2166 (31,3%)	
	Ensino superior	121/1375 (8,8%)	48/791 (6,1%)	169/2166 (7,8%)	
**Dados clínicos**					
**Atividade Física**					
	Sedentário	964/1470 (65,6%)	581/867 (67%)	1545/2337 (66,1%)	0,334
	Leve	419/1470 (28,5%)	246/867 (28,4%)	665/2337 (28,5%)	
	Moderada	79/1470 (5,4%)	39/867 (4,5%)	118/2337 (5%)	
	Intensa	8/1470 (0,5%)	1/867 (0,1%)	9/2337 (0,4%)	
Tabagismo Atual		109/1481 (7,4%)	73/873 (8,4%)	182/2354 (7,7%)	0,381
IMC (kg/m^2^) - média ± dp		29,2 ± 4,9 (n=1478)	28,9 ± 5 (n=873)	29,1 ± 5 (n=2351)	0,117
**Número de Comorbidades Presentes**					
	0	59/1482 (4%)	26/876 (3%)	85/2358 (3,6%)	0,257
	1	239/1482 (16,1%)	148/876 (16,9%)	387/2358 (16,4%)	
	2	670/1482 (45,2%)	372/876 (42,5%)	1042/2358 (44,2%)	
	3	514/1482 (34,7%)	330/876 (37,7%)	844/2358 (35,8%)	
**Comorbidades Presentes**					
	Hipertensão	1330/1482 (89,7%)	796/876 (90,9%)	2126/2358 (90,2%)	0,391
	Diabetes Mellitus tipo 2	642/1482 (43,3%)	401/876 (45,8%)	1043/2358 (44,2%)	0,247
	Dislipidemia	1149/1482 (77,5%)	685/876 (78,2%)	1834/2358 (77,8%)	0,72

Fonte: Próprio autor.

Dados expressos em média e desvio padrão ou frequência. IMC: índice de massa corporal.

**Figura 2:**
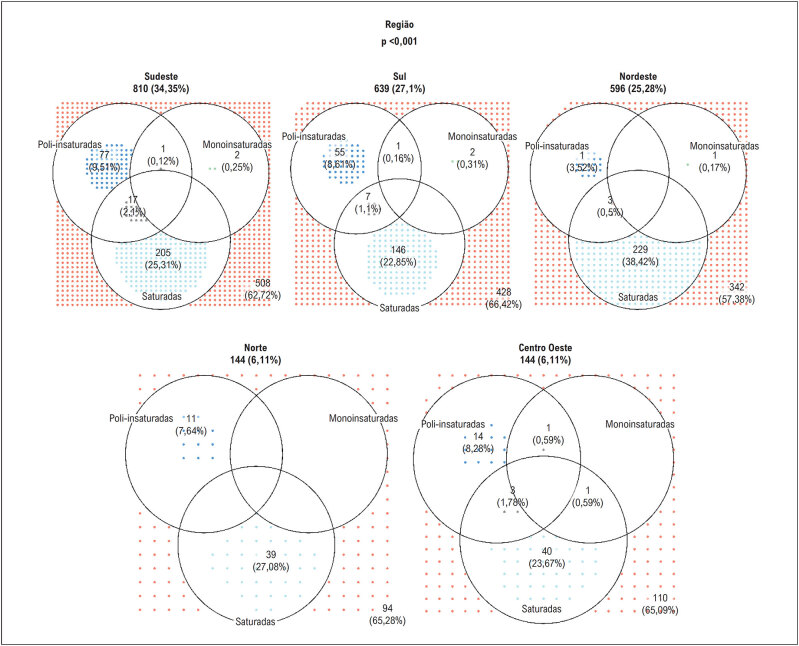
Distribuição da adesão às recomendações de ingestão de ácidos graxos de acordo com as regiões brasileiras.

Apesar de não se tratar de uma amostra representativa das cinco regiões brasileiras, avaliamos a hipótese de que a adesão às recomendações poderia ser diferente entre as regiões do país. Como é possível ver na Figura 2 , em todas as regiões a adesão a uma dieta com reduzida concentração de AGS foi maior que a adesão às demais recomendações. Entretanto, é observada uma associação entre adesão às recomendações e as regiões brasileiras, isto é, a adesão diferiu entre as regiões (p< 0,001). Na região Nordeste do país, observou-se uma maior proporção de pacientes que aderiram às recomendações de consumo de AGS quando comparada com as outras regiões. Por outro lado, a adesão às recomendações do consumo de AGPI foi menor.

**Figura 3:**
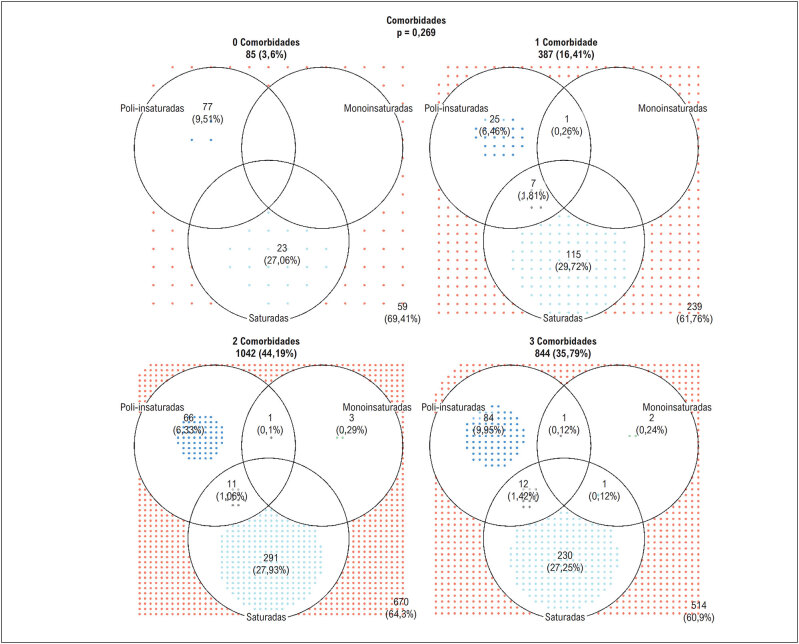
Representação gráfica da adesão às recomendações nutricionais de consumo de ácidos graxos de acordo com o número de fatores de risco cardiovascular.

Uma vez que recomendações nutricionais podem ser orientadas a partir da presença dos fatores de risco para DCV, verificou-se se a presença de múltiplas comorbidades poderia estar associada à adesão dessas recomendações, uma vez que o indivíduo pode ter recebido orientações para cada uma das comorbidades de forma individualizada ou ela foi intensificada pela presença de mais de um fator de risco. Na Figura 3 observamos, porém, que que não houve associação entre o número de comorbidades e a adesão às recomendações nutricionais (p = 0,269).

Considerando que alguma comorbidade poderia ter maior impacto na adesão às recomendações em detrimento à outra, foi avaliada a adesão segundo a presença de cada um dos fatores de risco separadamente (DM2, HAS e dislipidemia). Os resultados ( *dados não publicados* ) confirmam que a presença de qualquer comorbidade não influenciou a adesão, sendo ela igual à dos indivíduos sem alguma comorbidade específica.

Nas Tabelas 2 , 3 e 4 está descrito o consumo alimentar dos participantes de acordo com a adesão às recomendações dos diferentes ácidos graxos. Aqueles que atingiram as orientações de ingestão para AGS apresentaram menor consumo energético total da dieta, maior consumo de carboidratos e fibra alimentar e menor ingestão de sódio e colesterol dietético; por outro lado, aqueles que atingiram as recomendações de AGPI apresentaram menor consumo de carboidratos e maior consumo de sódio e fibras. Já entre os participantes com consumo adequado de AGM, observou-se menor ingestão de carboidratos e maior ingestão de colesterol na dieta.

**Tabela 2:** Distribuição da ingestão de macronutrientes de acordo com a adesão (sim ou não) às recomendações de ácidos graxos saturados

	Não adere à recomendação de AGS (n=1668)	Adere à recomendação de AGS (n=690)	Total (n=2358)	p
Energia (Kcal)	1408,3 [1115,5 - 1787,7]	1225,5 [979,2 - 1532,3]	1359,7 [1067,5 - 1714,6]	< **0,001**
Carboidrato (%)	50,6 [44,7 - 55,6]	60,5 [55,1 - 65,7]	53,2 [47 - 59,2]	< **0,001**
Proteína (%)	19,2 [16 - 23,1]	18,9 [15,1 - 23,2]	19,1 [15,7 - 23,1]	0,107
Lipídios (%)	29,4 [25,6 - 33,6]	19,8 [17,1 - 22,7]	26,9 [21,9 - 31,8]	< **0,001**
AGS (%)	9,9 [8,4 - 11,7]	5,7 [4,8 - 6,4]	8,7 [6,6 - 10,7]	< **0,001**
AGM (%)	8,7 [7,4 - 10,5]	5,4 [4,5 - 6,4]	7,8 [6,1 - 9,7]	< **0,001**
AGPI (%)	6,6 [5,2 - 8,3]	5,7 [4,5 - 7,1]	6,3 [4,9 - 8]	< **0,001**
Sódio (mg)	2634 [1947,5 - 3500,7]	2304 [1731,9 - 3040,1]	2545,5 [1880,3 - 3340,4]	< **0,001**
Colesterol dietético (mg)	193,6 [128 - 290,5]	111,2 [67,2 - 187,6]	168,9 [106 - 262,2]	< **0,001**
Fibra alimentar (g)	16,7 [11,5 - 23,9]	18,8 [13,8 - 26,4]	17,3 [12,2 - 24,5]	< **0,001**

Fonte: Próprio autor.

Dados expressos em mediana e intervalo interquartil. AGS: ácido graxos saturados; AGPI: ácidos graxos poli-insaturados; AGM: ácidos graxos monoinsaturados.

**Tabela 3:** Distribuição da ingestão de macronutrientes de acordo com a adesão (sim ou não) às recomendações de ácidos graxos poli-insaturados

	Não adere à recomendação de AGPI (n=2147)	Adere à recomendação de AGPI(n=211)	Total (n=2358)	p
Energia (Kcal)	1354 [1060,1 - 1715,4]	1391,4 [1091,2 - 1713,4]	1359,7 [1067,5 - 1714,6]	0,376
Carboidrato (%)	53,7 [47,7 - 59,7]	46,7 [40 - 53,1]	53,2 [47 - 59,2]	< **0,001**
Proteína (%)	19,2 [15,8 - 23,1]	18,8 [15,6 - 22,2]	19,1 [15,7 - 23,1]	0,293
Lipídios (%)	26,2 [21,4 - 31]	33,4 [29,5 - 38,7]	26,9 [21,9 - 31,8]	< **0,001**
AGS (%)	8,5 [6,5 - 10,7]	9,6 [8,1 - 11,6]	8,7 [6,6 - 10,7]	< **0,001**
AGM (%)	7,6 [5,9 - 9,5]	10 [8,3 - 12,3]	7,8 [6,1 - 9,7]	< **0,001**
AGPI (%)	6,1 [4,8 - 7,5]	11,5 [10,6 - 12,7]	6,3 [4,9 - 8]	< **0,001**
Sódio (mg)	2498,6 [1857,5 - 3297,6]	2832,9 [2230,6 - 3726]	2545,5 [1880,3 - 3340,4]	< **0,001**
Colesterol dietético (mg)	164,9 [104,2 - 253,9]	228,5 [145,7 - 352,5]	168,9 [106 - 262,2]	<0,001
Fibra alimentar (g)	17,2 [12,2 - 24,4]	18 [12,5 - 26,9]	17,3 [12,2 - 24,5]	0,2

Fonte: Próprio autor.

Dados expressos em mediana e intervalo interquartil. AGS: ácido graxos saturados; AGPI: ácidos graxos poli-insaturados; AGM: ácidos graxos monoinsaturados.

**Tabela 4:** Distribuição da ingestão de macronutrientes de acordo com a adesão (sim ou não) às recomendações de ácidos graxos monoinsaturados

	Não adere à recomendação de AGM (n=2349)	Adere à recomendação de AGM (n=9)	Total (n=2358)	p
Energia (Kcal)	1360,4 [1067,3 - 1714,9]	1324,4 [1077,3 - 1590,1]	1359,7 [1067,5 - 1714,6]	0,742
Carboidrato (%)	53,2 [47 - 59,2]	29,2 [21,9 - 50,3]	53,2 [47 - 59,2]	**0,001**
Proteína (%)	19,1 [15,8 - 23,1]	21,1 [14,8 - 26,2]	19,1 [15,7 - 23,1]	0,694
Lipídios (%)	26,8 [21,9 - 31,7]	46,4 [39,6 - 50,3]	26,9 [21,9 - 31,8]	< **0,001**
AGS (%)	8,7 [6,6 - 10,7]	17,1 [13,2 - 20,9]	8,7 [6,6 - 10,7]	< **0,001**
AGM (%)	7,8 [6,1 - 9,7]	22 [21,8 - 24,7]	7,8 [6,1 - 9,7]	< **0,001**
AGPI (%)	6,3 [4,9 - 8]	8 [7,6 - 10,8]	6,3 [4,9 - 8]	**0,004**
Sódio (mg)	2545,2 [1881,6 - 3340,8]	2743 [1583,2 - 3291,8]	2545,5 [1880,3 - 3340,4]	0,881
Colesterol dietético (mg)	168,8 [106 - 261,2]	500,6 [230,5 - 634]	168,9 [106 - 262,2]	0,024
Fibra alimentar (g)	17,3 [12,3 - 24,5]	10,2 [7,2 - 20,9]	17,3 [12,2 - 24,5]	0,073

Fonte: Próprio autor.

Dados expressos em mediana e intervalo interquartil. AGS: ácido graxos saturados; AGPI: ácidos graxos poli-insaturados; AGM: ácidos graxos monoinsaturados.

## Discussão

Neste estudo, a adesão às recomendações para ingestão de diferentes ácidos graxos dietéticos foi avaliada entre 2358 indivíduos em prevenção secundária para DCV com idade média de 63,2 ± 9 oriundos das cinco regiões brasileiras. Observou-se que nenhum indivíduo apresentou adesão a recomendação da ingestão dos três ácidos graxos (AGS, AGM e AGPI) de forma concomitante. A adesão às recomendações de ingestão do AGS foi a mais prevalente (27,95%) e a presença de diversas comorbidades não influenciou na adesão às recomendações.

A adesão à recomendação de ingestão inferior a 7% das calorias diárias proveniente de AGS, apesar de ser maior que a adesão às demais recomendações, ainda foi baixa na população avaliada. A recomendação de consumo de AGS para a população geral é de até 10% do CET diário. Nos Estados Unidos, observa-se que mais de 60% da população não atinge essa recomendação.^24^ No Brasil, o consumo médio de AGS pela população geral é de 8,9% do CET (intervalo de incerteza (UI) de 95% 7,5 a 10,3),^25^ percentual considerado adequado quando comparado às recomendações. Entretanto, o presente estudo mostra que a população brasileira especificamente em prevenção secundária para DCV apresenta um consumo médio de AGS superior ao esperado. Resultados similares foram identificados em outros estudos transversais com pacientes pós síndrome coronariana aguda nos quais o consumo médio de AGS foi de 9,3%^14^ e o percentual de indivíduos com consumo considerado adequado foi de aproximadamente 23%.^15^ Estima-se que limitar o consumo deste tipo de ácido graxo entre a população em geral esteja associado com a redução de 21% (risco relativo [RR] de 0,79; Intervalo de confiança de 95% [IC 95%] de 0,66 a 0,93) de eventos cardiovasculares combinados.^13^

Ao reduzir o consumo de AGS, outro macronutriente deve proporcionalmente aumentar para substitui-lo. Revisões sistemáticas com metanálises frequentemente apresentam resultados discrepantes referente ao efeito da redução do AGS na prevenção cardiovascular, por agregar artigos que diferem com relação à fonte dos nutrientes que substitui o AGS.^26^ Recomenda-se que a substituição seja feita por um ácido graxo insaturado;^25^ porém, como visto neste estudo, a população brasileira em prevenção cardiovascular secundária parece reduzir o AGS substituindo-o por carboidrato. A substituição do AGS por carboidrato não confere aumento no risco e ainda alguns autores discutem o tipo de carboidrato que estes estudos referem estar substituindo o AGS, podendo ter um efeito quando substituído por fontes alimentares ricas em fibras e outro quando substituído por fontes alimentares ricas em açúcares simples.^13^ A substituição do AGS por AGPI parece reduzir em 22% (RR =0,78; IC 95% 0,62 a 0,97) e o benefício da substituição por AGM ainda não está claro; ^13 , 26^ destaca-se que na amostra avaliada o pequeno percentual de indivíduos que atingiu as recomendações de ingestão de AGM só o fez em detrimento do aumento global da ingestão de gorduras totais, inclusive ultrapassando a recomendação de AGS.

Na população avaliada, AGPI foi consumido muito aquém ao esperado, reflexo do baixo consumo de alimento fonte desta gordura como peixes e óleos vegetais, como também é visto em outras populações (brasileiras e não) em prevenção secundária para DCV.^14 , 27^ O efeito pelo qual o AGPI confere proteção cardiovascular estaria associado à sua participação na redução do LDL-colesterol e na inflamação.^28^ Em 2010, o consumo inadequado de AGPI contribuiu com 711.800 (95% UI 680.700 a 745.000) mortes por causa cardiovascular, representando 10,3% (95% UI 9,9% a 10,6%) da mortalidade global, e o consumo inadequado de AGS contribuiu com 250.900 (95% UI 236.900 a 265.800), representando 3,6%, (95% UI 3,5% a 3,6%) da mortalidade global.^24^

Devido à interação in vivo dos macronutrientes na saúde, a avaliação dos componentes dietéticos por si só pode não ser um bom indicador de saúde. Concentrar-se nos padrões alimentares e considerar todos os aspectos da ingestão alimentar de um indivíduo fornece mais precisão para determinar o risco de doença. Com base em estudos anteriores, o padrão alimentar `ocidental’ foi associado a um maior risco de DCV e padrões alimentares como a dieta do Mediterrâneo e a dieta DASH vem sendo destacados e ganhando mais destaque nas diretrizes clínicas de prevenção cardiovascular.^29 , 30^ Exatamente por terem uma composição reduzida de AGS e alto em AGPI e AGM, além de outras características nutricionais como alto teor de fibras, antioxidantes e compostos bioativos, estes padrões alimentares saudáveis devem ser fortemente recomendados. Além disso, a orientação desses padrões de forma global parece ser mais factível para a compreensão do paciente (e consequentemente para a adesão) em comparação a simples recomendação para redução do consumo de alimentos fonte de AGS.^25^ Destaca-se que no momento da alta para o domicílio, entre indivíduos hospitalizados por um evento cardiovascular agudo, as orientações referentes a ingestão de nutrientes e de padrões alimentares parecem diferir de acordo com o sistema de saúde hospitalar: no Sistema Único de Saúde (realidade da maioria dos participantes alocados no estudo BALANCE) parecem prevalecer orientações para redução de gorduras, frituras e de sódio dietético; já no sistema privado, além dessas recomendações, prevalecem também orientações com relação ao consumo de lácteos, preparações culinárias grelhadas e cozidas no vapor, peixes, azeite de oliva extra virgem, frutas e verduras, e grãos integrais.^31^

Os resultados deste estudo enfatizam a baixa adesão dos participantes às recomendações estabelecidas para a ingestão de nutrientes. Podemos levantar a hipótese de que os pacientes em prevenção cardiovascular secundária enfrentam os seguintes desafios: a) acesso limitado a informações, devido à dificuldade para agendamento com profissionais de saúde especializados, à falta de orientações adequadas por parte dos profissionais de saúde devido à alta demanda (pouco tempo para atendimento) ou à falta de atualização; b) dificuldade de compreensão das orientações, pois traduzir as diretrizes em orientações práticas e compreensíveis não é uma tarefa simples; e c) dificuldade na adesão influenciada por questões financeiras, econômicas ou pela falta de acesso a alimentos frescos e minimamente processados. Independentemente das razões, é crucial entender esse cenário e propor estratégias para superar os desafios na mudança de comportamento alimentar. Nesse contexto, o BALANCE Trial buscou traduzir as recomendações nutricionais das diretrizes da SBC em orientações práticas que respeitassem os hábitos e a cultura alimentar regional. Essa estratégia foi publicada pelo Ministério da Saúde ( *Alimentação Cardioprotetora: Manual de orientações para profissionais de Saúde da Atenção Básica* ) e está amplamente disponível.^32^ No estudo BALANCE,^16^ após um seguimento de três anos, observou-se melhora na qualidade global da dieta, representada pelo aumento no consumo de vegetais e redução significativa no consumo de AGS. Essas recomendações, entretanto, não refletiram na redução de novos eventos cardiovasculares no período.

Nosso estudo possui algumas limitações como a não inclusão de uma amostra representativa por estado brasileiro, além do método de inquérito alimentar (recordatório alimentar de 24h) apresentar vieses como de memória e de sub relato. Além disso, trata-se de uma análise exploratória que não estava prevista originalmente no protocolo de pesquisa. Não coletamos informações específicas referentes a orientações nutricionais previamente recebidas pelos participantes; porém, acreditamos que os dados apresentados em nosso estudo destacam a importância de que, mesmo que tal orientação tenha sido realizada por algum profissional de saúde, ela pode não ter sido suficiente para mudar o comportamento alimentar do indivíduo. Nosso estudo também tem pontos fortes, como o tamanho amostral desta análise transversal e o cuidado metodológico como o treinamento minucioso para a coleta de dados oferecido às equipes de pesquisa de cada centro participante.

## Conclusão

Na amostra de brasileiros em prevenção secundária avaliada, identificou-se uma adesão insuficiente às recomendações nutricionais para o consumo de diferentes ácidos graxos. É importante ressaltar que nenhum dos participantes demonstrou adesão às recomendações de ingestão para os diversos ácidos graxos simultaneamente, reforçando a importância da implementação de estratégias de sensibilização para promover mudanças no estilo de vida nessa população.

## References

[1] Oliveira GMM, Brant LCC, Polanczyk CA, Malta DC, Biolo A, Nascimento BR (2022). Cardiovascular Statistics - Brazil 2021. Arq Bras Cardiol.

[2] (2023). Organização Pan-Americana de Saúde. Doenças cardiovasculares [Internet].

[3] Jernberg T, Hasvold P, Henriksson M, Hjelm H, Thuresson M, Janzon M. (2015). Cardiovascular Risk in Post-Myocardial Infarction Patients: Nationwide Real World Data Demonstrate the Importance of a Long-Term Perspective. Eur Heart J.

[4] Jortveit J, Halvorsen S, Kaldal A, Pripp AH, Govatsmark RES, Langørgen J. (2019). Unsatisfactory Risk Factor Control and High Rate of New Cardiovascular Events in Patients with Myocardial Infarction and Prior Coronary Artery Disease. BMC Cardiovasc Disord.

[5] Yusuf S, Joseph P, Rangarajan S, Islam S, Mente A, Hystad P (2020). Modifiable Risk Factors, Cardiovascular Disease, and Mortality in 155 722 Individuals from 21 High-Income, Middle-Income, and Low-Income Countries (PURE): A Prospective Cohort Study. Lancet.

[6] Rodríguez-Monforte M, Flores-Mateo G, Sánchez E. (2015). Dietary Patterns and CVD: A Systematic Review and Meta-Analysis of Observational Studies. Br J Nutr.

[7] Santos RD, Gagliardi AC, Xavier HT, Magnoni CD, Cassani R, Lottenberg AM (2013). First Guidelines on Fat Consumption and Cardiovascular Health. Arq Bras Cardiol.

[8] Faludi AA, Izar MCO, Saraiva JFK, Chacra APM, Bianco HT, Afiune A (2017). Atualização da Diretriz Brasileira de Dislipidemias e Prevenção da Aterosclerose – 2017. Arq Bras Cardiol.

[9] Avezum Á, Feldman A, Carvalho AC, Sousa AC, Mansur AP, Bozza AE (2015). V Guideline of the Brazilian Society of Cardiology on Acute Myocardial Infarction Treatment with ST Segment Elevation. Arq Bras Cardiol.

[10] Jones LK, Sturm AC, Gionfriddo MR (2022). Translating Guidelines Into Practice Via Implementation Science: An Update in Lipidology. Curr Opin Lipidol.

[11] Partridge MR (2003). Translating Research Into Practice: How are Guidelines Implemented?. Eur Respir J Suppl.

[12] Poli A. (2020). The PURE Study and the Enigmatic Aspects of the Diet: Is it Possible that an High Saturated Fat Consumption Would Not be Harmful?. Eur Heart J Suppl.

[13] Hooper L, Martin N, Jimoh OF, Kirk C, Foster E, Abdelhamid AS (2020). Reduction in Saturated Fat Intake for Cardiovascular Disease. Cochrane Database Syst Rev.

[14] Luz VC, Barbier SM, Portal VL, Stein E, Marcadenti A. (2021). Perfil Dietary and Anthropometric of Patients with Acute Coronary Syndrome Admitted to a Tertiary Hospital. Braspen J.

[15] Naud LM, Goulart AC, Santos ISS, Benesoenor IJM, Lotufo PA (2020). Assessment of Nutritional Status and Eating Habits of Patients with Acute Coronary Syndrome from the ERICO Project. Nut Brasil.

[16] Weber B, Bersch-Ferreira ÂC, Torreglosa CR, Marcadenti A, Lara ES, da Silva JT (2019). Implementation of a Brazilian Cardioprotective Nutritional (BALANCE) Program for Improvement on Quality of Diet and Secondary Prevention of Cardiovascular Events: A Randomized, Multicenter Trial. Am Heart J.

[17] Weber B, Bersch-Ferreira ÂC, Torreglosa CR, Ross-Fernandes MB, da Silva JT, Galante AP (2016). The Brazilian Cardioprotective Nutritional Program to Reduce Events and Risk Factors in Secondary Prevention for Cardiovascular Disease: Study Protocol (The BALANCE Program Trial). Am Heart J.

[18] Associação Brasileira de Empresas de Pesquisa (2012). Critério de Classificação Econômica Brasil.

[19] Matsudo S, Araújo T, Matsudo V, Andrade D, Andrade E, Oliveira LC (2001). Questionário Internacional de Atividade Física (IPAQ): Estudo de Validade e Reprodutibilidade no Brasil. Ativ Fis Saude.

[20] Barroso WKS, Rodrigues CIS, Bortolotto LA, Mota-Gomes MA, Brandão AA, Feitosa ADM (2021). Brazilian Guidelines of Hypertension - 2020. Arq Bras Cardiol.

[21] Moshfegh AJ, Rhodes DG, Baer DJ, Murayi T, Clemens JC, Rumpler WV (2008). The US Department of Agriculture Automated Multiple-Pass Method Reduces Bias in the Collection of Energy Intakes. Am J Clin Nutr.

[22] (2011). Universidade Estadual de Campinas. Núcleo de Estudos e Pesquisas em Alimentação. Tabela Brasileira de Composição de Alimentos – TACO [Internet].

[23] (2022). United States Department of Agriculture. Food Composition Database [Internet].

[24] Wang Q, Afshin A, Yakoob MY, Singh GM, Rehm CD, Khatibzadeh S (2016). Impact of Nonoptimal Intakes of Saturated, Polyunsaturated, and Trans Fat on Global Burdens of Coronary Heart Disease. J Am Heart Assoc.

[25] Van Horn L, Carson JA, Appel LJ, Burke LE, Economos C, Karmally W (2016). Recommended Dietary Pattern to Achieve Adherence to the American Heart Association/American College of Cardiology (AHA/ACC) Guidelines: A Scientific Statement from the American Heart Association. Circulation.

[26] Izar MCO, Lottenberg AM, Giraldez VZR, Santos RDD, Machado RM, Bertolami A (2021). Position Statement on Fat Consumption and Cardiovascular Health - 2021. Arq Bras Cardiol.

[27] Dardzińska JA, Małgorzewicz S, Szupryczyńska N, Gładyś K, Śliwińska A, Kaczkan M (2023). Adherence to the 2021 Dietary Guidelines of the European Society of Cardiology on Cardiovascular Disease Prevention in Residents of the Pomeranian Voivodeship with Increased Cardiovascular Risk. Pol Arch Intern Med.

[28] Ander BP, Dupasquier CM, Prociuk MA, Pierce GN (2003). Polyunsaturated Fatty Acids and their Effects on Cardiovascular Disease. Exp Clin Cardiol.

[29] Hou L, Li F, Wang Y, Ou Z, Xu D, Tan W (2015). Association between Dietary Patterns and Coronary Heart Disease: A Meta-Analysis of Prospective Cohort Studies. Int J Clin Exp Med.

[30] Yu E, Malik VS, Hu FB (2018). Cardiovascular Disease Prevention by Diet Modification: JACC Health Promotion Series. J Am Coll Cardiol.

[31] Lima TCRM, Silva DGD, Barreto IDC, Oliveira JC, Oliveira LCS, Arcelino LAM (2019). Quality of Intra-Hospital Nutritional Counseling in Patients with STEMI in the Public and Private Health Networks of Sergipe: The VICTIM Register. Arq Bras Cardiol.

[32] Brasil. Ministério da Saúde (2018). Alimentação Cardioprotetora: Manual de Orientações para os Profissionais de Saúde da Atenção Básica [Internet].

