# Supplementary material for: Adequação do Consumo de Ácidos Graxos entre Pacientes em Prevenção Cardiovascular Secundária
Source: Arq Bras Cardiol. 2024 Mar 20;121(3):e20230487. [Article in Portuguese] doi: 10.36660/abc.20230487 (PMC12094293; doi:10.36660/abc.20230487)
Supplement: Translation

# Translation: Adequacy of Fatty Acids Consumption Among Individuals in Secondary Cardiovascular Prevention

**DOI:** 10.36660/abc.20230487i

**Authors:** Aline Marcadenti, Rachel H. Vieira Machado, Renato Hideo Nakagawa Santos, Caio Cesar dos Santos Kasai, Cristiane Kovacs, Annie Bello, Cristina H. de Matos, Renata Torres Abib Bertacco, Gabriela C. Souza, Gabriela da S. Schirmann, Francisca Eugenia Zaina Nagano, Soraia Poloni, Raquel Milani El Kik, Naoel Hassan Feres, Isa G. Rodrigues, Antônio Carlos Sobral Sousa, Josilene M. F. Pinheiro, Sandra Mary Lima Vasconcelos, Daniele Maria de Oliveira Carlos, Viviane Sahade Souza, Adriana Barros Gomes, José Albuquerque de Figueiredo, Emilio Hideyuki Moriguchi, Maria Cristina Izar, Sônia Lopes Pinto, Josefina Bressan, Simone Raimondi de Souza, Magali C. Kumbier, Celme Barroncas Passos de Araújo, Camila R. Torreglosa, Bernardete Weber, Ângela Cristine Bersch-Ferreira

**Affiliations:** 1 São Paulo SP Brazil Instituto de Pesquisa do Hcor, São Paulo, SP – Brazil; 2 Londrina PR Brazil Pontifícia Universidade Católica do Paraná – Escola de Medicina Câmpus Londrina, Londrina, PR – Brazil; 3 São Paulo SP Brazil Instituto Dante Pazzanese de Cardiologia, São Paulo, SP – Brazil; 4 Rio de Janeiro RJ Brazil Instituto Nacional de Cardiologia, Rio de Janeiro, RJ – Brazil; 5 Itajaí SC Brazil Universidade do Vale do Itajaí, Itajaí, SC – Brazil; 6 Pelotas RS Brazil Universidade Federal de Pelotas, Pelotas, RS – Brazil; 7 Porto Alegre RS Brazil Universidade Federal do Rio Grande do Sul, Porto Alegre, RS – Brazil; 8 Bagé RS Brazil Universidade da Região da Campanha, Bagé, RS – Brazil; 9 Curitiba PR Brazil Universidade Federal do Paraná – Hospital de Clínicas, Curitiba, PR – Brazil; 10 Porto Alegre RS Brazil Hospital de Clínicas de Porto Alegre, Porto Alegre, RS – Brazil; 11 Porto Alegre RS Brazil Pontifícia Universidade Católica do Rio Grande do Sul, Porto Alegre, RS – Brazil; 12 Cuiabá MT Brazil Universidade Federal de Mato Grosso, Cuiabá, MT – Brazil; 13 Recife PE Brazil Pronto Socorro Cardiológico Universitário de Pernambuco, Recife, PE – Brazil; 14 Aracaju SE Brazil Universidade Federal de Sergipe, Aracaju, SE – Brazil; 15 Santa Cruz RN Brazil Hospital Universitário Ana Bezerra, Santa Cruz, RN – Brazil; 16 Maceió AL Brazil Universidade Federal de Alagoas, Maceió, AL – Brazil; 17 Fortaleza CE Brazil Hospital de Messejana, Fortaleza, CE – Brazil; 18 Salvador BA Brazil Universidade Federal da Bahia, Salvador, BA – Brazil; 19 São Luís MA Brazil Universidade Federal do Maranhão, São Luís, MA – Brazil; 20 São Paulo SP Brazil Universidade Federal de São Paulo (UNIFESP), São Paulo, SP – Brazil; 21 Palmas TO Brazil Universidade Federal do Tocantins, Palmas, TO – Brazil; 22 Viçosa MG Brazil Universidade Federal de Viçosa, Viçosa, MG – Brazil; 23 Rio de Janeiro RJ Brazil Instituto Estadual de Cardiologia Aloysio de Castro, Rio de Janeiro, RJ – Brazil; 24 Porto Alegre RS Brazil Consultoria Terapia Nutricional – COTENUT, Porto Alegre, RS – Brazil; 25 Manaus AM Brazil Hospital Universitário Francisca Mendes, Manaus, AM – Brazil; 26 São Paulo SP Brazil Real e Benemérita Associação Portuguesa de Beneficência, São Paulo, SP – Brazil

**Keywords:** Fatty Acids, Diet, Cardiovascular Disease, Secondary Prevention

## Abstract

**Background:**

Adhering to a diet adequate in macronutrients is crucial for the secondary prevention of cardiovascular diseases.

**Objective:**

To assess the prevalence of adherence to recommendations for the consumption of dietary fatty acids for the prevention and treatment of cardiovascular diseases and to estimate whether the presence of certain cardiovascular risk factors would be associated with adherence.

**Methods:**

Cross-sectional study using baseline data from 2,358 participants included in the "Brazilian Cardioprotective Nutritional Program Trial". Dietary intake and cardiovascular risk factors were assessed. Adequate intake of polyunsaturated fatty acids (PUFA) was considered as ≥10% of total daily energy intake; for monounsaturated fatty acids (MUFA), 20%; and for saturated fatty acids (SFA), <7% according to the Brazilian Society of Cardiology. A significance level of 5% was considered in the statistical analysis.

**Results:**

No participant adhered to all recommendations simultaneously, and more than half (1,482 [62.9%]) did not adhere to any recommendation. Adherence exclusively to the SFA recommendation was the most prevalent, fulfilled by 659 (28%) participants, followed by adherence exclusively to the PUFA (178 [7.6%]) and MUFA (5 [0.2%]) recommendations. There was no association between the number of comorbidities and adherence to nutritional recommendations (p = 0.269). Participants from the Brazilian Northeast region showed a higher proportion of adherence to SFA consumption recommendations (38.42%) and lower adherence to PUFA intake (3.52%) (p <0.001) compared to other regions.

**Conclusions:**

Among the evaluated sample, there was low adherence to nutritional recommendations for dietary fatty acid consumption.

## Introduction

Cardiovascular diseases (CVD) are considered the leading cause of morbidity and mortality in Brazil and worldwide, accounting for about seven million deaths annually. Particularly, CVD affects vulnerable groups such as the elderly, individuals with lower income, and limited education.^1 , 2^

Risk factors for CVD are targeted for the prevention of these diseases. However, greater attention in terms of treatment should be given to individuals undergoing secondary cardiovascular prevention, as a greater risk for the recurrence of cardiovascular events is established in this population.^3 , 4^These risk factors can be modifiable or non-modifiable; modifiable factors include hyperlipidemia, smoking, alcohol consumption, hyperglycemia, obesity, sedentary lifestyle, hypertension (HTN), and poor diet quality.^5^

Medical societies develop clinical guidelines regarding nutritional approaches to guide nutrient consumption and dietary patterns related to protection and risk for the development of CVD.^6^One of the nutritional recommendations highlighted in the guidelines refers to the importance of the proportion of different dietary fatty acids intake: saturated (SFA), monounsaturated (MUFA), polyunsaturated (PUFA), and trans-unsaturated (TFA).^7 - 9^The guidelines recommend that healthcare professionals advise their patients to have a dietary intake following healthy dietary patterns, including an adequate proportion of fatty acids; however, they do not describe how this guidance should be implemented. Additionally, it is necessary to adapt these guidelines to the cultural and financial context in which the individual is situated.^10 , 11^

The relationship between the consumption of different fatty acids, especially SFA, and the occurrence of cardiovascular events in the general population is still not established.^12 , 13^In terms of secondary cardiovascular prevention, little is known about the consumption of these nutrients,^14 , 15^and national-scale information on the adherence of these community-dwelling (non-hospitalized) patients to fatty acid consumption recommendations is not known. Thus, this study aimed to evaluate, at the baseline of a national multicenter randomized clinical trial, the prevalence of adherence to recommendations for dietary fatty acid consumption, as well as to estimate whether the presence of certain cardiovascular risk factors (dyslipidemia, HTN, or type 2 diabetes mellitus [T2DM]) was associated with adherence.

## Methods

This is an exploratory analysis of baseline data from participants in the "Brazilian Cardioprotective Nutritional Program Trial" - (BALANCE Program Trial, ClinicalTrials: NCT01620398). This was a multicenter study involving 2,534 individuals in secondary prevention for cardiovascular diseases from 35 centers distributed across the 5 regions of Brazil.^16^The randomized clinical trial aimed to evaluate the effectiveness of an educational nutritional intervention based on the nutritional recommendations advocated by Brazilian guidelines for the prevention of CVD on the prevention of new cardiovascular events.^16^

All eligibility criteria are reported in the study protocol.^17^Participants aged 45 years or older who presented one or more of the following established CVD in the last 10 years were included: coronary artery disease, stroke, and peripheral vascular disease. All volunteers read and signed the informed consent form before their final selection as research subjects. The Local Ethics Committees approved the study protocol.

### Data Collection

Trained interviewers administered a structured questionnaire containing questions about sociodemographic^18^and clinical characteristics. Participants were classified as current smokers, non-smokers, or ex-smokers, and the level of physical activity was categorized according to the International Physical Activity Questionnaire (IPAQ) short version.^19^Body Mass Index (BMI) was calculated using the formula body mass (kg)/height (m^2^). Blood pressure was obtained by a trained professional following the recommendations of the American Heart Association,^20^and medication data were obtained from medical prescriptions. Diagnoses of T2DM, HTN, and dyslipidemia were obtained from medical records. All data were recorded on an electronic case report form.

### Dietary Intake

Data on dietary intake were obtained through two 24-hour dietary recalls, and the average between the two recalls was used for the proposed analysis. Participants reporting dietary intake >4000 kcal/day or <500 kcal/day were excluded due to potential inconsistency in reporting. The Multiple Pass Method was used to standardize the collection of dietary intake data and to capture maximum information about consumed foods.^21^Dietary intake data were recorded using the Vivanda program (São Paulo, SP, Brazil), a Brazilian software that prioritizes Brazilian and American food composition tables.^22 , 23^A photo album containing images of standardized food portions, specifically prepared by the BALANCE Program Trial, was used to assist in the assessment of food intake. All researchers involved in data collection were trained both in obtaining information and using the software.

To verify if the participant achieved the recommendation for PUFA intake, a proportion equal to or greater than 10% of the total daily energy intake (TDEI) was considered.^7 , 8^The participant was considered to have achieved the recommendation for MUFA intake if they reported consumption equal to or greater than 20% of the TDEI.^8^Finally, to verify if the subject achieved the recommendation for SFA intake, an intake equal to or less than 7% of the TDEI was considered.^8 , 9^

### Statistical Analysis

The data were presented as absolute and relative frequencies for categorical variables and as position statistics (mean or median) and dispersion statistics (standard deviation and interquartile ranges) for continuous variables, depending on the normality of the data assessed by the Shapiro-Wilk test. Comparisons between participants who achieved at least one of the recommendations were conducted using Pearson's chi-square test or Fisher's exact test when appropriate for categorical variables and unpaired t-test for continuous variables. For macronutrient intake, patients who achieved a specific recommendation (SFA, MUFA, and PUFA intake) were compared using the non-parametric Mann-Whitney test. The Venn diagram was used to graphically represent the occurrence of simultaneous adherence to the recommendations for fatty acid intake. All analyses considered a two-tailed alpha of 5% and were performed using the R software (R Foundation for Statistical Computing, Vienna, Austria).

## Results

In the BALANCE study, 2,534 individuals in secondary prevention for cardiovascular disease were initially included; however, for the present analysis, only those with complete dietary records were considered. After excluding participants who reported dietary intake >4000 kcal or <500 kcal/day, the total sample of the study consisted of 2,358 individuals. Of these, 144 (6.1%) were from the Northern region of the country, 169 (7.2%) from the Midwest region, 596 (25.3%) from the Northeast region, 639 (27.1%) from the South region and 810 (34.5%) from the Southeast region ( Figure 1 ).

**Figure 1:** Distribution of participants included in the BALANCE study by national state.

Regarding the median percentages of fatty acid intake, it was identified as 8.7% (interquartile range 6.6 - 10.7) for SFA, 7.8% (6.1 - 9.7) for MUFA, and 6.3% (4.9 - 8.0) for PUFA. None of the individuals fully adhered to the recommendations for fatty acid consumption, and more than half (1,482 [62.9%]) did not adhere to any recommendation (Central Illustration). Adherence exclusively to the SFA recommendation was the most prevalent, being followed by 659 (28%) participants, followed by exclusive adherence to the PUFA recommendation (178 [7.6%]) and MUFA (5 [0.2%]). Additionally, 30 individuals (1.3%) adhered to both PUFA and SFA recommendations concurrently, three individuals to the PUFA and MUFA recommendations (0.1%), and only one individual (0.04%) to the MUFA and SFA recommendations simultaneously.

Table 1 describes the characteristics of participants who adhered to at least one recommendation (PUFA, MUFA, or SFA) and those who did not adhere to any of the recommendations. It is noteworthy that individuals from lower socioeconomic classes, according to the *Associação Brasileira de Empresas de Pesquisa* (ABEP) criteria and with lower education levels, showed greater adherence to the recommendations.

**Table 1:** Characteristics of participants adhering to any nutritional recommendation for fatty acid consumption and those not adhering to any recommendation

Features		Do not adhere to any recommendation (n=1482)	Adheres to at least 1 recommendation (n=876)	Total (n=2358)	p
**Demographic data**					
Age (years) - mean ± SD		63.3 ± 9.1	62.9 ± 8.7 (n=876)	63.2 ± 9	0.259
Female sex		606/1482 (40.9%)	374/876 (42.7%)	980/2358 (41.6%)	0.411
**Social class**					
	D/E	175/1375 (12.7%)	128/787 (16.3%)	303/2162 (14%)	*0.014*
	C	778/1375 (56.6%)	455/787 (57.8%)	1233/2162 (57%)	
	A/B	422/1375 (30.7%)	204/787 (25.9%)	626/2162 (29%)	
**Education**					
	Elementary School	803/1375 (58.4%)	515/791 (65.1%)	1318/2166 (60.8%)	*0.004*
	High School	451/1375 (32.8%)	228/791 (28.8%)	679/2166 (31.3%)	
	Higher Education	121/1375 (8.8%)	48/791 (6.1%)	169/2166 (7.8%)	
**Clinical data**					
**Physical Activity Level**					
	Sedentary	964/1470 (65.6%)	581/867 (67%)	1545/2337 (66.1%)	0.334
	Low	419/1470 (28.5%)	246/867 (28.4%)	665/2337 (28.5%)	
	Moderate	79/1470 (5.4%)	39/867 (4.5%)	118/2337 (5%)	
	Vigorous	8/1470 (0.5%)	1/867 (0.1%)	9/2337 (0.4%)	
Current Smoking		109/1481 (7.4%)	73/873 (8.4%)	182/2354 (7.7%)	0.381
BMI (kg/m^2^) - mean ± SD		29.2 ± 4.9 (n=1478)	28.9 ± 5 (n=873)	29.1 ± 5 (n=2351)	0.117
**Number of Comorbidities**					
	0	59/1482 (4%)	26/876 (3%)	85/2358 (3.6%)	0.257
	1	239/1482 (16.1%)	148/876 (16.9%)	387/2358 (16.4%)	
	2	670/1482 (45.2%)	372/876 (42.5%)	1042/2358 (44.2%)	
	3	514/1482 (34.7%)	330/876 (37.7%)	844/2358 (35.8%)	
**Comorbidities**					
	Hypertension	1330/1482 (89.7%)	796/876 (90.9%)	2126/2358 (90.2%)	0.391
	Type 2 Diabetes Mellitus	642/1482 (43.3%)	401/876 (45.8%)	1043/2358 (44.2%)	0.247
	Dyslipidemia	1149/1482 (77.5%)	685/876 (78.2%)	1834/2358 (77.8%)	0.72

Data are expressed as mean and standard deviation (SD) or frequency. BMI: body mass index.

Although this is not a representative sample of the five Brazilian regions, we evaluated the hypothesis that adherence to recommendations could differ among the regions of the country. As seen in Figure 2 , adherence to a diet with reduced SFA concentration was higher in all regions compared to adherence to other recommendations. However, an association between adherence to recommendations and Brazilian regions was observed, meaning that adherence differed among regions (p < 0.001). In the Northeast region of the country, a higher proportion of patients adhered to SFA consumption recommendations compared to other regions. On the other hand, adherence to PUFA consumption recommendations was lower.

**Figure 2:** Distribution of adherence to recommendations for dietary fatty acid intake according to Brazilian regions.

Since nutritional recommendations can be tailored based on the presence of risk factors for CVD, we investigated whether the presence of multiple comorbidities could be associated with adherence to these recommendations, as individuals may have received guidance for each comorbidity individually or it was intensified by the presence of more than one risk factor. In Figure 3 , however, we observed that there was no association between the number of comorbidities and adherence to nutritional recommendations (p = 0.269).

**Figure 3:** Graphical representation of adherence to nutritional recommendations for dietary fatty acid consumption according to the number of cardiovascular risk factors.

Considering that some comorbidity could have a greater impact on adherence to recommendations over another, adherence was evaluated according to the presence of each individual risk factor separately (T2DM, HTN, and dyslipidemia). The results (unpublished data) confirm that the presence of any comorbidity did not influence adherence, which was equal to that of individuals without any specific comorbidity.

In Tables 2 , 3 , and 4 , the dietary intake of participants is described according to adherence to recommendations for different fatty acids. Those who achieved the intake guidelines for SFA showed lower TDEI consumption, higher carbohydrate and dietary fiber consumption, and lower sodium and dietary cholesterol intake; on the other hand, those who achieved PUFA recommendations had lower carbohydrate consumption and higher sodium and dietary fiber intake. Meanwhile, among participants with adequate MUFA consumption, lower carbohydrate intake, and higher dietary cholesterol intake were observed.

**Table 2:** Distribution of macronutrient intake according to adherence (yes or no) to saturated fatty acid recommendations

	Does not adhere to the recommendation for SFA (n=1668)	adhere to the recommendation for SFA (n=690)	Total (n=2358)	p
Energy (kcal)	1408.3 [1115.5 - 1787.7]	1225.5 [979.2 - 1532.3]	1359.7 [1067.5 - 1714.6]	**<0.001**
Carbohydrate (%)	50.6 [44.7 - 55.6]	60.5 [55.1 - 65.7]	53.2 [47 - 59.2]	**<0.001**
Protein (%)	19.2 [16 - 23.1]	18.9 [15.1 - 23.2]	19.1 [15.7 - 23.1]	0.107
Total fats (%)	29.4 [25.6 - 33.6]	19.8 [17.1 - 22.7]	26.9 [21.9 - 31.8]	**<0.001**
SFA (%)	9.9 [8.4 - 11.7]	5.7 [4.8 - 6.4]	8.7 [6.6 - 10.7]	**<0.001**
MUFA (%)	8.7 [7.4 - 10.5]	5.4 [4.5 - 6.4]	7.8 [6.1 - 9.7]	**<0.001**
PUFA (%)	6.6 [5.2 - 8.3]	5.7 [4.5 - 7.1]	6.3 [4.9 - 8]	**<0.001**
Sodium (mg)	2634 [1947.5 - 3500.7]	2304 [1731.9 - 3040.1]	2545.5 [1880.3 - 3340.4]	**<0.001**
Dietary Cholesterol (mg)	193.6 [128 - 290.5]	111.2 [67.2 - 187.6]	168.9 [106 - 262.2]	**<0.001**
Dietary Fiber (g)	16.7 [11.5 - 23.9]	18.8 [13.8 - 26.4]	17.3 [12.2 - 24.5]	**<0.001**

Data are expressed as median and interquartile range. SFA: saturated fatty acids; PUFA: polyunsaturated fatty acids; MUFA: monounsaturated fatty acids.

**Table 3:** Distribution of macronutrient intake according to adherence (yes or no) to polyunsaturated fatty acids recommendations

	Does not adhere to the recommendation for PUFA (n=2147)	adhere to the recommendation for PUFA (n=211)	Total (n=2358)	p
Energy (kcal)	1354 [1060.1 - 1715.4]	1391.4 [1091.2 - 1713.4]	1359.7 [1067.5 - 1714.6]	0.376
Carbohydrate (%)	53.7 [47.7 - 59.7]	46.7 [40 - 53.1]	53.2 [47 - 59.2]	**<0.001**
Protein (%)	19.2 [15.8 - 23.1]	18.8 [15.6 - 22.2]	19.1 [15.7 - 23.1]	0.293
Total fats (%)	26.2 [21.4 - 31]	33.4 [29.5 - 38.7]	26.9 [21.9 - 31.8]	**<0.001**
SFA (%)	8.5 [6.5 - 10.7]	9.6 [8.1 - 11.6]	8.7 [6.6 - 10.7]	**<0.001**
MUFA (%)	7.6 [5.9 - 9.5]	10 [8.3 - 12.3]	7.8 [6.1 - 9.7]	**<0.001**
PUFA (%)	6.1 [4.8 - 7.5]	11.5 [10.6 - 12.7]	6.3 [4.9 - 8]	**<0.001**
Sodium (mg)	2498.6 [1857.5 - 3297.6]	2832.9 [2230.6 - 3726]	2545.5 [1880.3 - 3340.4]	**<0.001**
Dietary Cholesterol (mg)	164.9 [104.2 - 253.9]	228.5 [145.7 - 352.5]	168.9 [106 - 262.2]	**<0.001**
Dietary Fiber (g)	17.2 [12.2 - 24.4]	18 [12.5 - 26.9]	17.3 [12.2 - 24.5]	0.2

Data are expressed as median and interquartile range. SFA: saturated fatty acids; PUFA: polyunsaturated fatty acids; MUFA: monounsaturated fatty acids.

**Table 4:** Distribution of macronutrient intake according to adherence (yes or no) to monounsaturated fatty acids recommendations

	Does not adhere to the recommendation for MUFA (n=2349)	adhere to the recommendation for MUFA (n=9)	Total (n=2358)	p
Energy (kcal)	1360.4 [1067.3 - 1714.9]	1324.4 [1077.3 - 1590.1]	1359.7 [1067.5 - 1714.6]	0.742
Carbohydrate (%)	53.2 [47 - 59.2]	29.2 [21.9 - 50.3]	53.2 [47 - 59.2]	**0.001**
Protein (%)	19.1 [15.8 - 23.1]	21.1 [14.8 - 26.2]	19.1 [15.7 - 23.1]	0.694
Total fats (%)	26.8 [21.9 - 31.7]	46.4 [39.6 - 50.3]	26.9 [21.9 - 31.8]	**<0.001**
SFA (%)	8.7 [6.6 - 10.7]	17.1 [13.2 - 20.9]	8.7 [6.6 - 10.7]	**<0.001**
MUFA (%)	7.8 [6.1 - 9.7]	22 [21.8 - 24.7]	7.8 [6.1 - 9.7]	**<0.001**
PUFA (%)	6.3 [4.9 - 8]	8 [7.6 - 10.8]	6.3 [4.9 - 8]	0.004
Sodium (mg)	2545.2 [1881.6 - 3340.8]	2743 [1583.2 - 3291.8]	2545.5 [1880.3 - 3340.4]	0.881
Dietary Cholesterol (mg)	168.8 [106 - 261.2]	500.6 [230.5 - 634]	168.9 [106 - 262.2]	0.024
Dietary Fiber (g)	17.3 [12.3 - 24.5]	10.2 [7.2 - 20.9]	17.3 [12.2 - 24.5]	0.073

Data are expressed as median and interquartile range. SFA: saturated fatty acids; PUFA: polyunsaturated fatty acids; MUFA: monounsaturated fatty acids.

## Discussion

In this study, adherence to recommendations for the intake of different dietary fatty acids was evaluated among 2,358 individuals in secondary prevention for CVD with a mean age of 63.2 ± 9 years from the five Brazilian regions. It was observed that no individual adhered to the recommendation for the intake of all three fatty acids (SFA, MUFA, and PUFA) simultaneously. Adherence to SFA intake recommendations was the most prevalent (27.95%), and the presence of multiple comorbidities did not influence adherence to the recommendations.

Adherence to the recommendation of intake of <7% of daily calories from SFA, although higher than adherence to other recommendations, was still low in the evaluated population. The recommendation for SFA consumption for the general population is up to 10% of the daily total energy intake. In the United States, it is observed that over 60% of the population does not meet this recommendation.^24^In Brazil, the average consumption of SFA by the general population is 8.9% of the total energy intake (95% uncertainty interval [UI] 7.5 to 10.3),^25^a percentage considered adequate when compared to recommendations. However, this study shows that the Brazilian population, specifically in secondary prevention for CVD, presents a higher average consumption of SFA than expected. Similar results were identified in other cross-sectional studies with post-acute coronary syndrome patients, where the average consumption of SFA was 9.3%,^14^and the percentage of individuals with considered adequate consumption was approximately 23%.^15^It is estimated that limiting the consumption of this type of fatty acid among the general population is associated with a 21% reduction (relative risk [RR] of 0.79; 95% confidence interval [CI] 0.66 to 0.93) in combined cardiovascular events.^13^

When reducing SFA consumption, another macronutrient should proportionally increase to replace it. Systematic reviews with meta-analyses often present discrepant results regarding the effect of reducing SFA on cardiovascular prevention, as they aggregate articles that differ in terms of the source of nutrients replacing SFA.^26^It is recommended that an unsaturated fatty acid make that replacement;^25^however, as seen in this study, the Brazilian population in secondary cardiovascular prevention seems to reduce SFA by replacing it with carbohydrates. The replacement of SFA with carbohydrates does not confer an increased risk, and some authors discuss the type of carbohydrate that these studies refer to when replacing SFA, which may have an effect when replaced by fiber-rich food sources and another when replaced by sources rich in simple sugars.^13^The substitution of SFA with PUFA seems to reduce by 22% (RR = 0.78; 95% CI 0.62 to 0.97), and the benefit of substitution with MUFA is still unclear.^13 , 26^It should be noted that in the evaluated sample, the small percentage of individuals who met the recommendations for MUFA intake only did so at the expense of an overall increase in total fat intake, including exceeding the SFA recommendation.

In the evaluated population, PUFA was consumed well below expectations, reflecting the low consumption of foods rich in this fat, such as fish and vegetable oils, as also seen in other populations (Brazilian and non-Brazilian) in secondary prevention for CVD.^14 , 27^The protective cardiovascular effect conferred by PUFA would be associated with its participation in reducing LDL cholesterol and inflammation.^28^In 2010, inadequate PUFA consumption contributed to 711,800 (95% UI 680,700 to 745,000) cardiovascular deaths, representing 10.3% (95% UI 9.9% to 10.6%) of global mortality, and inadequate SFA consumption contributed to 250,900 (95% UI 236,900 to 265,800), representing 3.6% (95% UI 3.5% to 3.6%) of global mortality.^24^

Due to the *in vivo* interaction of macronutrients in health, the assessment of dietary components alone may not be a good health indicator. Focusing on dietary patterns and considering all aspects of an individual's dietary intake provides more accuracy in determining disease risk. Based on previous studies, the `Western’ dietary pattern has been associated with a higher risk of CVD. In contrast, dietary patterns such as the Mediterranean diet and the DASH diet have been highlighted and are gaining more attention in cardiovascular prevention clinical guidelines.^29 , 30^These healthy dietary patterns should be strongly recommended because they have a reduced composition of SFA and are high in PUFA and MUFA, in addition to other nutritional characteristics such as high fiber content, antioxidants, and bioactive compounds. Furthermore, guidance on these patterns globally seems to be more feasible for patient understanding (and consequently for adherence) compared to simple recommendations to reduce the consumption of SFA-rich foods.^25^It should be noted that at the time of discharge to home, among individuals hospitalized for an acute cardiovascular event, the guidance regarding nutrient intake and dietary patterns seems to differ according to the hospital healthcare system: in the Unified Health System (the reality of most participants allocated in the BALANCE study), guidance for reducing fats, fried foods, and dietary sodium seems to prevail; in the private system, in addition to these recommendations, guidance regarding dairy consumption, grilled and steamed culinary preparations, fish, extra virgin olive oil, fruits and vegetables, and whole grains also prevail.^31^

The results of this study underscore the low adherence of participants to established recommendations for nutrient intake. We can hypothesize that patients in secondary cardiovascular prevention face the following challenges: a) limited access to information due to difficulty scheduling appointments with specialized healthcare professionals or inadequate guidance from healthcare professionals due to high demand (limited time for consultations) or lack of updates; b) difficulty understanding the guidance, as translating guidelines into practical and understandable recommendations is not a simple task; and c) adherence difficulties influenced by financial, economic issues, or lack of access to fresh and minimally processed foods. Regardless of the reasons, it is crucial to understand this scenario and propose strategies to overcome the challenges in dietary behavior change. In this context, the BALANCE Trial sought to translate the nutritional recommendations from the Brazilian Society of Cardiology guidelines into practical guidance that respects regional dietary habits and culture. This strategy was published by the Ministry of Health (Cardioprotective Nutrition: Guidance Manual for Basic Healthcare Professionals) and is widely available.^32^In the BALANCE study,^16^after a three-year follow-up, an improvement in the overall diet quality was observed, represented by an increase in vegetable consumption and a significant reduction in SFA consumption. However, these recommendations did not reflect in the reduction of new cardiovascular events during the period.

Our study has some limitations, such as the lack of inclusion of a representative sample from each Brazilian state, as well as the method of dietary assessment (24-hour dietary recall), which may introduce biases such as memory and underreporting. Additionally, it is an exploratory analysis that was not originally planned in the research protocol. We did not collect specific information regarding previous nutritional guidance received by participants; however, we believe that the data presented in our study highlight the importance that, even if a healthcare professional provided such guidance, it may not have been sufficient to change the individual's dietary behavior. Our study also has strengths, such as the sample size of this cross-sectional analysis and the methodological care taken, including thorough training provided to the research teams at each participating center for data collection.

## Conclusion

In the sample of Brazilians in secondary prevention evaluated, insufficient adherence to nutritional recommendations for the consumption of different fatty acids was identified. It is important to note that none of the participants demonstrated adherence to intake recommendations for various fatty acids simultaneously, emphasizing the importance of implementing awareness strategies to promote lifestyle changes in this population.
